# Personality disorder: a disease in disguise

**DOI:** 10.1080/03009734.2018.1526235

**Published:** 2018-12-12

**Authors:** Lisa Ekselius

**Affiliations:** Department of Neuroscience, Psychiatry, Uppsala University, Sweden

**Keywords:** ICD-11, personality disorders, personality traits, review article

## Abstract

Personality disorders (PDs) can be described as the manifestation of extreme personality traits that interfere with everyday life and contribute to significant suffering, functional limitations, or both. They are common and are frequently encountered in virtually all forms of health care. PDs are associated with an inferior quality of life (QoL), poor health, and premature mortality. The aetiology of PDs is complex and is influenced by genetic and environmental factors. The clinical expression varies between different PD types; the most common and core aspect is related to an inability to build and maintain healthy interpersonal relationships. This aspect has a negative impact on the interaction between health-care professionals and patients with a PD. From being discrete and categorical disease entities in previous classification systems, the current concept of PD, reflected in the newly proposed ICD-11, is a dimensional description based on the severity of the disturbed functioning rather than on the type of clinical presentation. Insight about the characteristics of PDs among medical practitioners is limited, which is partly because persons do not seek health care for their PD, but instead for other medical issues which are obscured by their underlying personality problems. What needs to be emphasized is that PDs affect both the clinical presentation of other medical problems, and the outcome of these, in a negative manner and that the integrated effects of having a PD are a shortened life expectancy. Accordingly, PDs need to be recognized in clinical practice to a greater extent than previously.

## Introduction

In everyday clinical practice persons who think, feel, behave, or relate to others differently than the average person are identified. This deviation from the norm is a central feature in all personality disorders (PDs). Although using slightly different formulations over the years, PDs are roughly characterized by ‘a pervasive pattern of thought, feeling and behaviour that characterize an individual’s unique lifestyle and mode of adaptation, which deviates markedly from the expectations of the individual’s culture’ (1). Such characteristics obviously create problems for those who bear them. PDs are likely to have an onset in adolescence or early adulthood, appear to be stable over time, and lead to impairment or distress (1,2).

This review, which is an overview on PDs and the core problems these ultimately lead to, is commenced with some background information about the concept of personality and on the attempts that have been made to understand and to describe different characteristics of personality, how these characteristics can be structured and understood, and about the deviations in normal personality that form the basis for the different types of PD. Above all, the paper focuses on problems met in primary and specialist health care. Such problems are common, and persons with PDs are known to be under-treated with respect to physical health (3) and are over-represented in the group categorized as the ‘difficult patient’ (4,5).

The present paper argues that all health-care professionals need basic knowledge about manifestations of different personality traits, above all in the form of manifest PDs, as we know that such pathology has a negative effect on the interaction between the patient and health-care professionals in terms of communication, clinical assessment, treatment, and outcome (6). The patients’ suffering is considerable, and generally they report a low QoL (7,8). Having a PD also infers a risk factor for premature mortality (9,10), which affects individuals and incurs a high cost to society (11).

## A historic perspective of aberrant personalities

The large variation in the way individuals think, feel, and behave has been recognized throughout antiquity. The terms for these characteristics have been diverse. For instance, Confucius (551–479 BCE) used the combination of ‘blood and vital essence’. The Greek philosopher and naturalist Theophrastus (c. 371 to c. 287 BC) used the term ‘characters’, and in eighteenth-century France the Galenus–Hippocrates term ‘temperament’ was reinstituted. The term ‘personality’ has been used since the eighteenth century to label distinguishing qualities of a person (12).

Pathological personalities have also generated interest over the years. Since the fourth century BC, philosophers have been trying to understand what it is that makes ‘us’ what we are. Theophrastus, a scholar of Plato and Aristotle, was the first to publish a systematic description of the multifaceted nature of personality types (12). A few hundred years later, Aelius Galenus (130–200 AD) linked Hippocrates’ four humours to personality characteristics in his description of *sanguine*, *phlegmatic*, *choleric*, and *melancholic* temperaments. He proposed that each of these four body fluids held a combination of two properties split along two axes: temperature (hot/cold) and humidity (wet/dry). The humoral pathology system influenced the view among European doctors until the breakthrough of medical science in the nineteenth century.

In the early nineteenth century, Franz Joseph Gall (1758–1828), a German neuroanatomist, thought that some brain areas were associated with specific functions. He also thought that measurements of the skull represented differences in the individual’s personality (13).

Philippe Pinel (1745–1826), a French physician, was the first to include an *aberrant* personality in the nosology of psychiatry (14). Pinel introduced the term ‘*manie sans délire*’ (mania without delusion). During that time, the term ‘mania’ was employed to refer to states of agitation. Pinel described a few of his male patients who were disposed to bursts of irrational anger and impulsive violence in response to minor irritation. In the same intellectual environment Jean-Étienne Dominique Esquirol (1772–1840) introduced the concept *monomanie raisonnante* and the Englishman James Cowles Prichard (1786–1848) used the term *moral insanity*. These three physicians were obsessed by the practical question at that time whether psychiatry could explain abnormal behaviour in persons lacking acute psychiatric symptoms who had committed a violent crime (14).

During the late nineteenth and early twentieth century, several conceptual systems for normal and abnormal personalities emerged as the result of the work of European psychologists and psychiatrists (e.g. Ribot, Heymans, Lazursky, Schneider, and Sjöbring).

Théodule Ribot (1839–1916), a French psychologist, described normal and abnormal characters. He pointed out that a person’s character is stable from childhood into adult life. Ribot described three primary personality types: the sensitive, the active, and the apathetic, all three of which were divided into subtypes (15).

The Dutch scientist Gerard Heymans (1857–1930) applied empirical methods to the study of personality. He developed the Cube of Heymans, a description of a personality typology. He defined personality types in three dimensions: ‘activity level’, ‘emotionality’, and ‘primary versus secondary functioning’, with the last-mentioned dimension comparable to ‘extroversion/introversion’. These three dimensions are represented on the *x*-, *y*-, and *z*-axes of the Heymans cube, where all combinations of the three dimensions defined eight personality types (16).

The contributions of Aleksandr Lazursky (1874–1917), a Russian psychologist, were not widespread because most of his publications were in Russian and because of the political climate of the time. His major contribution was the description of the ‘endopsychic’ and ‘exopsychic’ aspects of personality. The endopsychic components represent the psychological functions (e.g. perception, memory, attention, thinking) that are mainly inborn; the exopsychic components are the consequence of the interaction with the outside world. The interplay between these two aspects of personality determines how a person functions in an integrated social context (17).

The German psychiatrist Kurt Schneider (1887–1967) focused on diagnostic issues that included concepts of ‘psychopathy’, which he had broadly equated to PDs. Based on his clinical views (18), he vaguely defined abnormal personality as a statistical deviation from the norm. He proposed 10 psychopathic personalities, all of which are very similar to those in the current classifications of PDs in the DSM-5 and ICD-10 (19).

Henrik Sjöbring (1879–1956), a Swedish psychiatrist, suggested four constitution factors of the personality: capacity (intelligence), validity (psychic energy), stability (balance in keynote), and solidity (firmness, tardiness, tenacity). By these variables, all persons can be categorized as either normal, super-, or sub-: e.g. subcapable (unintelligent), subvalid (lack of psychic energy), normosolid, superstable, and so on (20).

The first modern attempt to determine the structure of human personality was credited to the English scientist Sir Francis Galton (1882–1911). He used a lexical approach to the dimensions of personality based on the assumption that those personality characteristics important to a group of people will eventually be represented in their language (21). This work was continued by several others (22), and the lexical hypothesis constitutes the basis for how current approaches describe personality dimensions. It is also important to mention the work of the psychologists Raymond Bernard Cattell (1905–1998) (23) and Gordon Willard Allport (1897–1967) (24) who independently used advanced statistics (e.g. factor analysis) to discern dimensions of personality.

## Modern concepts of personality disorder and personality

Before discussing this issue, it needs to be re-emphasized that the description of personalities is based purely on observations, or rather expressions, of the individual’s way to think, feel, behave, or relate. As a corollary, it follows that PDs are diagnoses based on symptoms described by the persons themselves, by persons in their surroundings, or are objectively observed in study situations. This circumstance accounts for why the validity and reliability of the current diagnostic instruments lack optimality (25).

Current knowledge on pathological personalities is primarily based on studies from four perspectives, all of which are necessary to create an in-depth template of what characterizes personality pathology.

The first perspective is the clinical picture, i.e. the integrated presentation of the clinical symptoms that are either expressed or witnessed. This perspective is what constitutes the basis for the clinical structured diagnosis according to classification systems. The second perspective entails a determination of underlying dysfunctional personality traits as well as dysfunctional limitations on capacity and functionality in the brain’s cognitive, emotional, and impulse control systems. The third perspective relates to the brain’s biological systems and their functions; this third perspective has highly benefited from the rapid development of brain imaging techniques (26). The fourth perspective denotes the underlying genetic contribution to the above-mentioned phenomena (27), which is currently approached in whole-genome association studies (28).

Not unexpectedly, studies have shown that the aetiology of personality pathology is complex. Overwhelming evidence supports the idea that an interaction between genetic and environmental factors is necessary for the development of human personality. The relation between the dimensions of normal personality and PD is not clear, however. Even if a PD has been viewed as an overexpression of personality traits to the extent that they lead to clinically significant distress or impairment, it has recently been demonstrated that a moderate-to-sizable proportion of the genetic influence underlying PD is not shared with the domain constructs of normative personality (29).

Based on the hypothesis that the domains of dysfunction in PDs are linked to specific neural circuits, neuroimaging techniques have been used over the past decade to examine the neural integrity of these circuits in personality-disordered individuals. Currently, the literature is flooded with information acquired through this approach. Most studies are done to explore borderline PD (30). In general, the studies have thus far demonstrated deviations in neuronal circuitry in areas previously found to be active in the symptomatology that characterizes the specific type of PD. Even if the results of such studies contribute to an understanding of underlying physiological processes, they are not yet ready to be used in clinical practice.

Several studies have examined the effects of being exposed to childhood adversities and the risk to develop PD. Just to mention one such study, we recently showed that exposure to cumulative childhood adversity was incrementally associated with a diagnosis of PD in young adulthood (31). Furthermore, childhood or adolescent psychiatric disorders have been suggested to trigger a chain of behaviours and responses that foster the more persistent psychopathology of a PD (32,33).

To determine the importance of genetic and environmental factors in early childhood in personality pathology the relationship between vulnerability to child abuse and antisocial personality patterns in adulthood was investigated (34). It was shown that individuals with a gene polymorphism that resulted in a low activity in monoamine oxidase A (MAOA) were more vulnerable to developing an antisocial personality pattern than those who had high activity in the MAOA gene, given that they had been exposed to child abuse. This gene–environment interaction has subsequently been confirmed (35). Moreover, a similar interaction for the effects of child maltreatment on antisocial behaviour has also been shown for other genes (36,37).

There is thus reason to consider genetic and environmental factors as interacting systems of crucial importance in the development of functional and dysfunctional personality traits.

## Classification of personality disorders

The differences in the types of aberration in thought, feeling, and behaviour have been the basis for the classification of different PDs. The characteristics described by Galenus, and later by e.g. Pinel and Schneider, are very similar to contemporary classification systems. What today are referred to as PDs were earlier called ‘pathological personalities’ or ‘*persona pathologica*’ and were found under that heading in earlier versions of the ICD (up to ICD-8). These diagnoses were used rarely, in part because of their stigmatizing connotations.

Up to now, classification of PDs has been based on fulfilling a specified number of defined and ‘specific’ criteria for each PD, resulting in a categorical description; if a defined number of these criteria were met, a disorder was acknowledged, else not.

Over time, there has been an intraprofessional dispute on whether the classification of PDs should be based on the defined and specific characteristics or on the severity of the functional aberration. Historically, and currently, in the ICD-10 (which is from 1992) and in the current American DSM-5 (38) (from 2013) classification is based on types of symptom, i.e. characteristics of the clinical presentation, represented by the abovementioned ‘specific’ criteria for each PD. ICD-10 describes nine discrete and specific (as well as one unspecified) types of PD (Table 1). The DSM-5 (38) identifies 10 PDs of similar structure. The DSM system has gone one step further in classification by grouping the different disorders in three clusters based on some overall common features. To illustrate, cluster A contains odd and eccentric personalities, cluster B includes dramatic, impulsive, emotional personalities, and cluster C fearful and anxious personalities.

**Table 1.** Personality disorders in the ICD-10 (2).

Code	Disorder	Characteristics in brief
F60.0	Paranoid	Excessive sensitivity to setbacks, unforgiveness of insults, recurrent suspicions without justification regarding the sexual fidelity of the spouse or sexual partner, and a combative and tenacious sense of personal rights.
F60.1	Schizoid	Withdrawal from affectional, social, and other contacts, preference for fantasy, solitary activities, and introspection. Limited capacity to express feelings and to experience pleasure.
F60.2	Dissocial	Disregard for social obligations, callous unconcern for the feelings of others. Gross disparity between behaviour and prevailing social norms. Behaviour not readily modifiable by adverse experience, including punishment. Low tolerance to frustration; low threshold for discharge of aggression, including violence; tendency to blame others, all leading to conflict with society.
F60.3	Emotionally unstable	A tendency to act impulsively and without consideration of the consequences; unpredictable and capricious mood. Liability to outbursts of emotion and incapacity to control the behavioural explosions. Tendency to quarrelsome behaviour and to conflicts with others. Two types are distinguished: the impulsive type with emotional instability and lack of impulse control; and the borderline type, with added disturbances in self-image, aims, and internal preferences, chronic feelings of emptiness, intense and unstable interpersonal relationships, and a tendency to self-destructive behaviour, including suicide gestures and attempts.
F60.4	Histrionic	Shallow and labile affectivity, self-dramatization, theatricality, exaggerated expression of emotions, suggestibility, egocentricity, self-indulgence, lack of consideration for others, easily hurt feelings, and continuous seeking for appreciation, excitement, and attention.
F60.5	Anankastic	Feelings of doubt, perfectionism, excessive conscientiousness, checking and preoccupation with details, stubbornness, caution, and rigidity. There may be insistent and unwelcome thoughts or impulses that do not attain the severity of an obsessive-compulsive disorder.
F60.6	Anxious [avoidant]	Feelings of tension and apprehension, insecurity and inferiority. A continuous yearning to be liked and accepted, hypersensitivity to rejection and criticism with restricted personal attachments, and a tendency to avoid certain activities by habitual exaggeration of the potential dangers or risks in everyday situations.
F60.7	Dependent	Pervasive passive reliance on other people to make one’s major and minor life decisions, great fear of abandonment, feelings of helplessness and incompetence, passive compliance with the wishes of elders and others, and a weak response to the demands of daily life. Lack of vigour may show itself in the intellectual or emotional spheres; often a tendency to transfer responsibility to others.
F60.8	Other specific	Eccentric, ‘haltlose’ type, immature, narcissistic, passive-aggressive, psychoneurotic.
F60.9	Unspecified	Diffuse symptoms, not fully qualifying for specific PD, but with the general criterion fulfilled.

A basic feature common for the different classification systems is that the aberration must be severe enough to cause a functional impairment in everyday life. This is the ‘general criterion’ for all PDs and overrides other perspectives. In other words, even the observance of very odd behaviour or feelings is not enough for a clinical diagnosis of a PD unless it can be ascertained that they lead to impairment or distress in everyday life.

Currently, there is somewhat of a paradigm shift in that more and more arguments speak for the relevance of a dimensional classification of PD based on the severity of symptoms rather than on the specific characteristics (19). Studies have, thus, shown that the conceptualization of PDs into discrete categories results in an insufficient description of the problem. Rather, it seems that within each discrete PD category the level of dysfunction is dimensional and dependent on the number of criteria fulfilled (39). Furthermore, only about half of all individuals with diagnosable PD fulfil criteria for a specific PD and are thus given a diagnosis of unspecified PD (40). In addition, the expression of different symptoms evolves continuously across the lifespan (41).

## General description of personality disorders in ICD-11

The new ICD-11 classification system, which was released by WHO in June 2018 (42), means a radical change in the classification of PDs and will impact all aspects of health care, as well as influence the way PDs are seen (43). After 1–2 years of adaptive work, ICD-11 is expected to be operative internationally in 2020–2021. In ICD-11 PDs have been classified based on the perspectives mentioned above, i.e. according to the severity of suffering, and are divided into three severity groups: *mild*, *moderate*, or *severe*. The degree of severity is determined by the extent of problems in interpersonal relationships or the ability and willingness to perform expected social and occupational roles, or both (see Table 2 for a full description).

**Table 2.** Personality disorders in the forthcoming ICD-11.

6D10 Personality disorder			
Description	Personality disorder is characterized by problems in functioning of aspects of the self (e.g. identity, self-worth,accuracy of self-view, self-direction), and/or interpersonal dysfunction (e.g. ability to develop and maintain close and mutually satisfying relationships, ability to understand others’ perspectives and to manage conflict in relationships) that have persisted over an extended period of time (e.g. 2 years or more). The disturbance is manifested in patterns of cognition, emotional experience, emotional expression, and behaviour that are maladaptive (e.g. inflexible or poorly regulated) and is manifested across a range of personal and social situations (i.e. is not limited to specific relationships or social roles). The patterns of behaviour characterizing the disturbance are not developmentally appropriate and cannot be explained primarily by social or cultural factors, including socio-political conflict. The disturbance is associated with substantial distress or significant impairment in personal, family, social, educational, occupational, or other important areas of functioning.		
	6D10.0	6D10.1	6D10.2
	Mild	Moderate	Severe
Description	All general diagnostic requirements for Personality Disorder are met. Disturbances affect some areas of personality functioning but not others (e.g. problems with self-direction in the absence of problems with stability and coherence of identity or self-worth) and may not be apparent in some contexts. There are problems in many interpersonal relationships and/or in performance of expected occupational and social roles, but some relationships are maintained, and/or some roles carried out. Specific manifestations of personality disturbances are generally of mild severity. Mild Personality Disorder is typically not associated with substantial harm to self or others but may be associated with substantial distress or with impairment in personal, family, social, educational, occupational, or other important areas of functioning that is either limited to circumscribed areas (e.g. romantic relationships, employment) or present in more areas but milder.	All general diagnostic requirements for Personality Disorder are met. Disturbances affect multiple areas of personality functioning (e.g. identity or sense of self, ability to form intimate relationships, ability to control impulses and modulate behaviour). However, some areas of personality functioning may be relatively less affected. There are marked problems in most interpersonal relationships and the performance of most expected social and occupational roles are compromised to some degree. Relationships are likely to be characterized by conflict, avoidance, withdrawal, or extreme dependency (e.g. few friendships maintained, persistent conflict in work relationships and consequent occupational problems, romantic relationships characterized by serious disruption or inappropriate submissiveness). Specific manifestations of personality disturbance are generally of moderate severity. Moderate Personality Disorder is sometimes associated with harm to self or others, and is associated with marked impairment in personal, family, social, educational, occupational, or other important areas of functioning, although functioning in circumscribed areas may be maintained.	All general diagnostic requirements for Personality Disorder are met. There are severe disturbances in functioning of the self (e.g. sense of self may be so unstable that individuals report not having a sense of who they are or so rigid that they refuse to participate in any but an extremely narrow range of situations; self-view may be characterized by self-contempt or be grandiose or highly eccentric). Problems in interpersonal functioning seriously affect virtually all relationships, and the ability and willingness to perform expected social and occupational roles is absent or severely compromised. Specific manifestations of personality disturbance are severe and affect most, if not all, areas of personality functioning. Severe Personality Disorder is often associated with harm to self or others and is associated with severe impairment in all or nearly all areas of life, including personal, family, social, educational, occupational, and other important areas of functioning.
6D11 Prominent personality traits or patterns			
Description	Trait domain qualifiers may be applied to Personality Disorders or Personality Difficulty to describe the characteristicsof the individual’s personality that are most prominent and that contribute to personality disturbance. Trait domainsare continuous with normal personality characteristics in individuals who do not have Personality Disorder orPersonality Difficulty. Trait domains are not diagnostic categories, but rather represent a set of dimensions thatcorrespond to the underlying structure of personality. As many trait domain qualifiers may be applied as necessary todescribe personality functioning. Individuals with more severe personality disturbance tend to have a greater numberof prominent trait domains.		
6D11.0	Negative affectivity in personality disorder or personality difficulty		
6D11.1	Detachment in personality disorder or personality difficulty		
6D11.2	Dissociality in personality disorder or personality difficulty		
6D11.3	Disinhibition in personality disorder or personality difficulty		
6D11.4	Anankastia in personality disorder or personality difficulty		
6D11.5	Borderline pattern		

Excerpt from reference (41) with permission from WHO.

A new feature in ICD-11 is the introduction of the term ‘Personality Difficulty’, which refers to pronounced personality characteristics that may affect treatment or health services but do not rise to the level of severity to merit a diagnosis of PD.

## Dimensions of personality disorders in ICD-11

ICD-11 has, thus, wiped out all type-specific categories of PD apart from the main one, the presence of PD itself. Instead, the type of clinical manifestation is added as a specific ‘post-coordination’ code describing the clinical characteristics in the form of six different personality domains. Factor analytic strategies have supported five domains, although clinical reasoning has suggested six domains (44–46). These six clinically derived personality domains do not fully correspond with the different specific PD types in the earlier ICD-10 (Table 2).

The domain traits are not inherently pathological, but rather represent a profile of underlying personality structure (19). They apply equally to individuals without any PD and to those with severe disorder, but in PD they show where the focus of the disorder is apparent. In severe disorder, several domain traits are likely to be associated with the disorder (47). To describe personality functioning, as many domains as necessary can be applied.

The descriptions of the six different domain traits below are slightly and only linguistically modified from those in the original ICD-11.

### Negative affectivity in personality disorder

The core aspect of negative affectivity is the tendency to experience a broad range of negative emotions. Common manifestations, not all of which may be present in everyone at a given time, include experiencing a variety of negative emotions with a frequency and intensity out of proportion to the situation: emotional lability and poor emotion regulation, negativistic attitudes, low self-esteem, low self-confidence, and mistrustfulness.

Patients fulfilling criteria for this disorder were classified as anxious/avoidant in previous classifications.

### Detachment in personality disorder

The core aspect of the detachment domain is the tendency to maintain interpersonal (social detachment) and emotional distance (emotional detachment). Common manifestations, not all of which may be present in everyone at a given time, include social detachment (avoidance of social interactions, lack of friendships, and avoidance of intimacy) and emotional detachment (reserve, aloofness, and limited emotional expression and experience).

This disorder type is like the schizoid type of PD described in ICD-10.

### Dissocial or antisocial personality disorder

Dissocial or antisocial PD is characterized by a gross disparity between behaviour and the prevailing social norms as well as by a callous unconcern for the feelings of others. Moreover, this type of PD can be described by a number of other attributes, including a gross and persistent attitude of irresponsibility and disregard for social norms, rules, and obligations; an incapacity to maintain enduring relationships, although having no difficulty in establishing them; very low tolerance to frustration and a low threshold for discharge of aggression, including violence; an incapacity to experience guilt or to profit from experience, particularly punishment; and finally, a marked proneness to blame others or to offer plausible rationalizations for the behaviour that has brought the person into conflict with society.

Persons with a dissocial PD often have an early criminal record and exhibit conduct problems in childhood or adolescence. The construct of a dissocial PD largely overlaps characteristics of the concept of psychopathy, which is not a term used to define a psychiatric disorder (48).

### Disinhibition in personality disorder

The core aspect of disinhibition traits is the tendency to act rashly based on immediate external or internal stimuli (i.e. sensations, emotions, thoughts) without consideration of the consequences. Common manifestations—not all of which may be present in everyone at a given time—include impulsivity, distractibility, irresponsibility, recklessness, and lack of planning.

Patients fulfilling the criteria for this disorder in previous classifications were classified as histrionic, narcissistic, or borderline.

### Anankastia in personality disorder

Anankastic PD (or obsessive-compulsive PD) is characterized by a narrow focus on orderliness and perfectionism and on right and wrong, although it also implies a need to control one’s own behaviour and the behaviour of others, as well as the need to control one’s environment to ensure conformity to these standards. Common manifestations, not all of which may be present in a given individual at a given time, can include conscientiousness (e.g. concern with social rules, obligations, and norms of right and wrong, scrupulous attention to detail, rigid, systematic, day-to-day routines, obsessiveness about hyper-scheduling, emphasis on organization, orderliness, and neatness) and emotional and behavioural constraint (e.g. rigid control over emotional expression, stubbornness and inflexibility, risk-avoidance, perseveration, and deliberativeness).

### Borderline personality disorder

The criteria for this disorder are very similar to those for disinhibition. It was included in the new ICD-11 at a very late stage of the process (45). The classification may be applied to individuals whose pattern of personality disturbance is characterized by a pervasive pattern of instability of interpersonal relationships, self-image, and affects, as well as marked impulsivity, as indicated by many of the following behavioural patterns: frantic efforts to avoid real or imagined abandonment; a pattern of unstable and intense interpersonal relationships; identity disturbance, manifested in markedly and persistently unstable self-image or sense of self; a tendency to act rashly in states of high negative affect, leading to potentially self-damaging behaviours; recurrent episodes of self-harm; emotional instability due to marked reactivity of mood; chronic feelings of emptiness; inappropriate intense anger or difficulty controlling anger; and transient dissociative symptoms or psychotic-like features in situations of high affective arousal. The condition involves anxiety without an identifiable connection to concrete stimuli and, among other things, has been called ‘annihilation anxiety’, ‘pan-anxiety’, or ‘global anxiety’. The term ‘emptiness depression’ describes general feelings of despair and hopelessness with dominance of depressive thoughts.

Borderline PD (in ICD-10 ‘emotionally unstable PD’) is a dominating diagnosis in out- and inpatient psychiatric care (9,10). Clinical expressions are more evident in younger ages and tend to decrease with advancing age.

Borderline PD is associated with high mortality by suicide (9,10,49). There is also a high risk to die prematurely because of impulsive risk-taking, as well as succumbing to violence from others (9,10). Recurrent suicidal threats or attempts, when combined with fears of abandonment, are strongly indicative of the diagnosis (50). Even if these characteristics make borderline pattern PD easy to identify, the diagnosis is often overlooked. A key reason for this neglect is the perception that the overemotional, sometimes theatrical, and self-injurious behaviours are signs of wilfulness and manipulations rather than signs of an illness (51).

Borderline PD occasionally includes depressive and anxiety symptoms and mild irritability. In general, many persons with borderline PD describe recurrent occasions with panic anxiety, which may lead to suspicion of a primary panic disorder or generalized anxiety disorder. Likewise, experienced social discomfort and fears can arouse suspicion of primary social anxiety disorder. Finally, recent studies have suggested that attention deficit hyperactivity disorder (ADHD), bipolar disorder, and borderline PD have similar origins or share common pathological mechanisms (52,53).

## Prevalence and longitudinal perspective

PDs are common in the general population. A recent overview (54), based on 13 studies conducted in the USA and Europe, reported prevalence figures varying from 3.9% to 15.5%. In the WHO World Mental Health Survey, carried out in 13 countries, the prevalence rate was 6.1% (55). The large variation in prevalence may be due to differences in sampling, diagnostic methods, and study settings. Furthermore, there may be differences in culture regarding significant personality pathology.

Persons in contact with the health-care system exhibit higher prevalence of PDs as compared with those not in contact. In fact, one-fourth of patients in primary care (56) and about half of those in psychiatric outpatient facilities fulfil the criteria for a PD (57).

PDs are equally common or more common in men (54) in the general population. In clinical settings, however, PDs are more often recognized in women, probably due to the higher rates of help-seeking behaviour in women (9,10).

Stability over time has long been a basic concept both in the description of personality and of PDs. Supporting this concept is the observation that there is rank-order stability over time in the expression of personality symptoms (58). On the other hand, an exaggeration in some personality traits over the life course and a decline in others have been observed (41,59). Furthermore, it has recently been shown that drugs affecting serotonin uptake can modulate personality traits (60,61). There is less support for the idea that a PD should be regarded as stable over time. Actually, modern research has shown that, although a maladaptive personality can be recognized early in life, it evolves continuously across the lifespan and is more plastic than previously believed (41). In addition, in the case of coexisting mental disorders, their contribution to the clinical picture will vary over time with the state of these disorders. In other words, even if personality traits are largely constant across time, there is a tendency that symptoms in persons with a PD change more over time than those without a PD. This change is often in the direction of improvement (62,63).

## Diagnosis, differential diagnosis, and psychiatric comorbidity

Establishing a formal diagnosis of a PD is an issue for specialist psychiatry, where it must be regarded as a time-process function. The patient history must cover the life perspective to understand the current clinical landscape in context and against a background of the individual’s unique developmental history.

General and permanent problems in work, studies, and relationships are often primary and obvious observations. Difficulties in interpersonal relations are often visible already at the first patient encounter. Those difficulties justify a step-by-step deepening of the formal diagnostic work while initiating treatment efforts. Enhanced personal knowledge will also provide a more nuanced image of the patient’s problems as well as adaptive resources.

Accounts of the current problem and the patient’s current life situation are a natural starting point when collecting data on the clinical history of the patient. Special attention must be given to the risks of suicide and violence. The clinical history should be expanded in a piecemeal manner on appropriate occasions.

During the diagnostic process, it often becomes clear that the patient presents with criteria for several disorders, both within and outside the PD spectrum. Such comorbidity is common (64,65); it is seen across the whole spectrum of PDs and other mental disorders and in general represents a broader pattern of symptoms as well as a more severe condition. This is reflected in the observation that the total number of fulfilled criteria for any PD is related to the observed dysfunction and to the reported QoL (54). Comorbidity between PDs and other mental disorders contributes significantly to functional impairment (64) while also increasing the risk of early mortality (9,10).

Because of this characteristic, it is not uncommon that the person who fulfils criteria for a diagnosis of PD will seek health care for another mental disorder, a fact that might be misleading during the diagnostic process. Not too infrequently, there are rapid onset depressive or anxiety states that motivate the care episode during which the coexisting problems related to a comorbid PD are apparent. A more pressing issue is comorbidity of a more enduring character. For example, a coexisting ADHD can obscure the clinical symptoms of borderline PD. Conversely, a severe and prolonged eating disorder may dominate over an underlying personality pathology.

When the symptomatic picture of PD is complex and partially overlapping between different diagnoses, it is seldom possible to distinguish between different underlying pathologies. The differential diagnostic procedure will therefore be more about evaluating the relative influence of the various demonstrable expressed symptoms on the severity of functioning.

To optimize diagnostic accuracy self-assessment tools, semi-structured interviews and personality inventories can be used. The SCID-II is such an interview support for personality diagnosis according to the DSM-IV and DSM-5 (66).

## Personality disorders and health

The long-term negative effects of having a PD are significant (9,10,41,54). Furthermore, because a PD is often overlooked diagnostically, the potential risks for the bearer may go undetected. A certain proportion of those who fulfil the criteria for a diagnosis of PD will ultimately have psychiatric care, while almost everyone eventually comes in contact with primary care or specialized somatic care. Given that personality-related problems lead to varying degrees of lack of adaptivity in interaction with other people, there are often complications in the contacts with health care and social services.

Personality traits are well known to impact health-related QoL (8) and outcome in health care. The negative consequence of having a high degree of neuroticism has been extensively studied (67). The consequence of having a PD is, however, only well studied in psychiatric care, where a multitude of studies have shown that having a comorbid PD represents a more severe condition with a worse prognosis as stated above.

There has been less focus on somatic care. Still, it has been shown that having a PD is related to higher rates of pain, greater use of analgesics, and more primary care, taken together suggesting an increase in somatic morbidity (68). It has also been shown that the outcome of treatment for somatic ill health is usually worse in the presence of a PD (68). For instance, having a PD was found to increase the risk of stroke (69) and coronary artery disease (70,71). Furthermore, having any PD is strongly associated with severe occupational outcomes, including disability benefits, regardless of disability diagnosis (72–74).

The most studied PD in this respect is borderline PD. Persons with this PD tend to be impulsive, and where self-harm is common they have an increased tendency to seek health care (75). Borderline PD, however, is related to an increased risk for several health problems, and consequently for a greater consumption of health care (76–79).

Based on previous knowledge that individuals with a PD have a higher mortality rate and a shorter life expectancy compared with the general population (69,80–83), we recently assessed to what extent this was related to type of PD or cause of death (9,10). Data from nationwide Swedish hospital registers with a follow-up of 25 years were used. Overall, all-cause standardized mortality ratios (SMRs) were found to increase in all clusters of PDs for natural and unnatural causes of death. The overall SMR was 6.1 in women and 5.0 in men, figures in line with those previously reported for anorexia nervosa, with higher rates in cluster B and unspecified/other PDs. The increased mortality ratio was seen for all somatic causes of death, reflecting the impact of having a PD on somatic health and wellbeing. The SMR for suicide was as high as 34.5 in women and 16.0 in men for cluster B disorders. Somatic and psychiatric comorbidity increased SMRs further. This excess mortality was also observed for most patients diagnosed with PDs not severe enough to lead to hospitalization (9). This observation contradicts the idea that only those persons with a PD severe enough to motivate inpatient treatment are burdened by an increased risk in mortality. This has important practical implications in that most patients with clinical problems linked to a PD are only treated as outpatients. On the other hand, the risk of death in those only treated as outpatients was clearly less than in those who received inpatient care, supporting the view of a difference in clinical severity between these groups (47).

## Aspects on handling and treatment

Given the impact of PDs on treatment outcomes in somatic and mental health care, a clinical pattern suggesting the existence of such a disorder should be identified in primary or somatic specialist care (84). Treatment is a concern for specialist psychiatry, however. A well-developed liaison psychiatry, a subspecialty of psychiatry, is particularly suitable.

Even if treatment modalities are not the topic of this survey, some basic principles must be addressed. Because PDs are deeply ingrained ways of thinking and behaving that evolved as the personality developed, they are considered difficult to treat. In recent years several studies have emerged that have, to some extent, changed this concept (85–88). In general, there are many challenges and no simple solution in the treatment of PDs. At the same time, because fundamental problems of PDs are related to interpersonal relations, a structured and stable relationship between the patient and the clinician is the basis for any successful approach. This ‘therapeutic alliance’ looms as the strongest predictor of successful outcome of any treatment attempt. The most difficult challenge for the clinician is to achieve this goal. In fact, the pre-existing quality of the patient’s relationships, rather than the type of PD, is the single factor that most affects the quality of this alliance (89). The strength of this alliance is crucial not only to obtain anamnestic information about the true history of the problems encountered but also to motivate and maintain adherence to treatment.

Although there is only a paucity of randomized controlled studies on the effect of psychotherapy in PD (90), the few studies published suggest that it should be the core treatment (91), leading to individual benefit and a reduction in care costs (92).

Currently, no pharmaceuticals are registered for use in PDs. Any attempt to apply a pharmacological approach is therefore an issue for the psychiatric specialist. Such an approach should aim to reduce or eliminate specific symptoms seen in other psychiatric disorders, where there is evidence that the drug in question is efficient. Irrespective of which drug is used, the clinical effect should be closely monitored.

The underlying hypothesis when attempting psychotropic pharmaceuticals in the treatment of PDs is the assumption that the features and attributes associated with the clinical expression are linked to biochemical abnormalities and thus can be regulated by psychotropic drugs. For most specific PDs, studies on the putative benefit of pharmaceuticals are lacking, and where studies have been done the results are at best modest (93). Despite this limitation, most psychiatrists can testify about patients with PDs who are prescribed many drugs, often over a long time, and without information about the expected or obtained benefit, or how the treatment was followed up. Such polypharmacy, particularly in combination with poor documentation, can put patients at risk of adverse drug events (side effects) and interactions. In other words, drugs should never be the first-line treatment but may be justified as a supplement to other treatment forms in specific situations.

## Conclusion

PD frequently goes undetected, in the shade of other health problems or diseases. PD is a predictor of worse health, premature death, and more serious life issues. It constitutes a challenge to health-care professionals and, above all, a burden for the patient, the family, and society. PD involves deviations in cognition, affectivity, interpersonal functioning, and/or impulse control. Deepened knowledge requires intellectual approaches based on sociodemographic, as well as epidemiological and advanced genetic and imaging techniques.

There is a clinical shift from an earlier focus on the characteristics of discrete PD entities to an awareness of the common features of different PDs, the suffering of patients, and the many problems they face in interpersonal relationships and daily life. The new ICD-11 classification aims to improve the description of the severity of problems encountered by patients.

Knowledge of the clinical aspects of PDs in general health care, vigilance to symptoms of PD, and appropriate diagnosis are all essential for optimal support to affected patients.

## Disclosure statement

No potential conflict of interest was reported by the authors.

## Note on the contributor

Lisa Ekselius, MD, PhD, is a Full Professor of Psychiatry at Uppsala University and a Senior Consultant in Psychiatry at the Uppsala University Hospital. Her research is focused on issues related to personality and personality disorders. These include the epidemiology of personality disorders, but also the contribution of personality traits to the expression of somatic as well as psychiatric disorders, and to the vulnerability to recover from major body trauma.
